# Evaluation of Psychometric Properties of Hardiness Scales: A Systematic Review

**DOI:** 10.3389/fpsyg.2022.840187

**Published:** 2022-06-01

**Authors:** Hamid Sharif Nia, Erika Sivarajan Froelicher, Lida Hosseini, Mansoureh Ashghali Farahani

**Affiliations:** ^1^School of Nursing and Midwifery, Mazandaran University of Medical Sciences, Sari, Iran; ^2^Department of Physiological Nursing, School of Nursing, University of California, San Francisco, San Francisco, CA, United States; ^3^Department of Epidemiology and Biostatistics, School of Medicine, University of California, San Francisco, San Francisco, CA, United States; ^4^School of Nursing and Midwifery, Iran University of Medical Sciences, Tehran, Iran

**Keywords:** hardiness, hardy personality, systematic review, psychometric testing, validation studies, validity, reliability

## Abstract

**Background:**

Hardiness is one of the personality traits that can help individuals in stressful situations. Since human beings are constantly under stressful situations and the stresses inflicted on people in each situation are different, various scales have been developed for assessing this feature among different people in different situations. Hence, it becomes necessary for researchers and health workers to assess this concept with valid and reliable scales. This systematic review aims to rigorously assess the methodological quality and psychometric properties of hardiness scales.

**Method:**

In the first step, the databases including Scopus, PubMed, Web of science, and Persian databases were searched using suitable keywords without limitation time. We select eligible suitable studies after screening titles and abstracts. The quality of studies was evaluated using the Consensus-based Standards for the selection of health Measurement Instruments (COSMIN) checklist and the Terwee quality criteria.

**Result:**

Of the 747 articles identified, 33 articles were entered in this study. Based on the COSMIN checklist, the most reported properties were as following structural validity (84%), hypothesis testing (56%), content validity (42%), and internal consistency (39%). Furthermore, 12 studies reported cross-cultural validity, three studies criterion validity, and one study reported measurement error.

**Conclusion:**

The “family caregivers’ hardiness scale,” “Japanese Athletic Hardiness Scale,” “Occupational Hardiness Questionnaire,” and “Children’s Hardiness Scale” are the best tools for assessing hardiness in family caregivers, athletes, employees, and children respectively. In addition, the “Dispositional Resilience Scale” (DRS-15) and The Personal Views Survey (PVS III-R) are the most frequently used scales with suitable features for measuring hardiness in the general population.

## Introduction

Human beings are constantly growing and moving from one stage to the other. This personal development process is an unpredictable and demanding process during each of the development stages during stressful circumstances (Maddi, 2004; Sharif Nia et al., 2021). These stress conditions can have a negative effect on performance, motivation, and health if they are not handled well (Bonanno, 2004). It should be noted that in addition to the natural and continuous stresses during the growth process, the current circumstances create conditions that add additional stresses by rapid changes in all spheres of life (Efimova et al., 2019). Many people cannot control these stressful situations. This in turn can threaten the individual’s physical, mental, and social aspects of their health (Bigalke, 2015).

Hardiness is one personality trait that can help individuals in stressful situations. The concept of hardiness was first proposed by Kobasa in 1979 based on the existence theory, which is conceptualized as one of the main personality structures for understanding motivation, excitement, and behavior (Kobasa, 1979). This concept finds meaning in the face of stressful situations are considered as a buffered and intervening variable that moderates the relationship between stressful situations and the physical and psychological effects (Abdollahi et al., 2018). Hardiness is a combination of attitudes and beliefs that motivate an individual to do hard and strategic work in the face of stressful and difficult situations (Maddi, 2007). Kobasa defined hardiness as a multidimensional personality trait consisting of three components or the 3C’s: commitment, control, and challenge (Kobasa, 1979). Commitment was defined as a tendency to engage in life’s activities and to have a genuine interest and curiosity about the world around us (activities, things, and others) and it includes a feeling of personal competence and feeling of community and/or corporation, control was defined as believing and acting as if one can influence the events of one’s life, and this belief in influence occurs as part of one’s efforts. This feature allows the person to perceive the predictable consequences of their activities in stressful events and manage them favorably (Luceño-Moreno et al., 2020). Finally, the tendency to challenge was defined as the belief that change, rather than stability, as a natural way of life creates opportunities for personal growth rather than a threat to one’s security (Kobasa, 1979).

It should be noted that in 2005, Maddi proposed another dimension called connection as the fourth dimension or the 4th C of hardiness (Maddi and Khoshaba, 2005). According to him, individuals gain part of their power and ability to face stressful situations because of communication with other members of society. Therefore, communication is one of the factors that play an important role in creating and maintaining hardiness (Maddi and Khoshaba, 2005). In 2017 Mund proposed culture as the fifth dimension or the 5th C influencing hardiness. In other words, she proposed that hardiness should not be interpreted as a simple approach regardless of culture (Mund, 2017).

Hardiness is a trait that is related to the person and his environment. Because the prevailing social and cultural conditions affect a person’s perception and experience of hardship and threat. In addition, his/her understanding of protective factors and how to use them, and through this, the hardiness dimensions and meanings can be formed (Chan, 2000; Benishek et al., 2005; Green et al., 2020). Therefore, by examining this concept in different groups of people with different stressful situations, various definitions, and components of it have been proposed according to the target community and the context and situation of stressful situations (Hosseini et al., 2021). For example, occupational hardiness means endurance and ability in difficult situations and in fact refers to a person’s performance based on cognitive assessments (Moreno-Jiménez et al., 2014). Wagnild and Young also conducted studies on the concept of hardiness in older women and concluded that the meaning of this concept in this group of people includes: equanimity, self-efficacy, perseverance, meaningfulness, and existential aloneness (Wagnild and Young, 1988). Likewise, because hardiness can be taught to people, in order to improve this feature and the ability of people to deal with stressful situations and reduce the effects of stress. Different scales have been developed for different groups such as college students, children, nursing students, and managers (Bartone, 1991; Benishek et al., 2005; Moreno-Jiménez et al., 2014). It should be noted that knowing the degree of the hardiness of individuals or evaluating the effectiveness of interventions requires an accurate and valid scale with desirable psychometric properties (Hosseini et al., 2021). Importantly, these scales consist of different dimensions, and some scales do not cover all the dimensions of hardiness. Hence, this systematic review aims to evaluate the psychometric properties of these scales and make recommendations about their use.

## Methods

### Study Design

This is a systematic review to evaluate the psychometric properties of the hardiness scales that were conducted according to the Preferred Reporting Items for Systematic Reviews and Meta-Analyses (PRISMA) guidelines (Moher et al., 2009).

### Eligibility Criteria

Eligibility criteria of this study included English and Persian articles describing the psychometric properties of scales/the process of validation/cross-cultural evaluation of the concept of hardiness. Excluded were articles with irrelevant topics, review/systematic review articles, structural equation model or model testing articles, and articles in languages other than Persian and English.

### Information Sources

Five electronic databases such as Scopus, PubMed, Science Direct, ProQuest, and Web of Science were searched for English articles. Two Persian databases including Persian SID^1^ and MAGIRAN^2^ were also searched for Persian articles. Finally, Google Scholar as a search engine and ProQuest database were searched to identify relevant theses. It is noteworthy that the reference lists of all identified articles were also searched manually. The search took place from the years 1979–2022.

### Search Strategy Electronic

The search strategy was based on the principle that considering a wide range of search terms leads to the best results of related studies. Therefore, in this study, the search strategy was designed taking into account the main concept, which is hardiness, and the type of study, which includes development or psychometric studies and using considering “abstract, title and keywords.” These keywords were used: hardiness, hardy personality, personality hardiness validity, validation, reliability, development, and psychometric. The Persian meanings of these keywords were used for searching in Persian databases. It is noteworthy that each database was searched with proper syntaxes (see Table 1).

**TABLE 1:** Keywords used in the search for the different databases.

Databases	Search string
PubMed	(((((((validity[Title/Abstract]) OR (validation[Title/Abstract])) OR (reliability[Title/Abstract])) OR (”Factor analysis”[Title/Abstract])) OR (psychometric[Title/Abstract])) OR (development[Title/Abstract])) AND (((hardiness[Title/Abstract]) OR (hardy personality[Title/Abstract])) OR (personality hardiness[Title/Abstract]))) AND ((((((((scale[Title/Abstract]) OR (survey[Title/Abstract])) OR (questionnaire[Title/Abstract])) OR (index[Title/Abstract])) OR (Inventor[Title/Abstract])) OR (Test [Title/Abstract])) OR (Measure[Title/Abstract])) OR (Instrument[Title/Abstract])) 187
Scopus	(TITLE-ABS-KEY [“validity” OR “validation” OR “reliability” “development” OR “psychometric”] AND TITLE-ABS-KEY [“hardiness” OR “hardy personality” OR “personality hardiness”]) 77
Web of science	(validity OR validation OR reliability OR “Factor analysis” OR psychometric OR development) AND (“hardiness” OR “hardy personality” OR “personality hardiness”)AND (scale OR survey OR questionnaire OR index OR Inventor OR Test OR Measure OR Instrument) 187

### Study Selection

**FIGURE 1:**
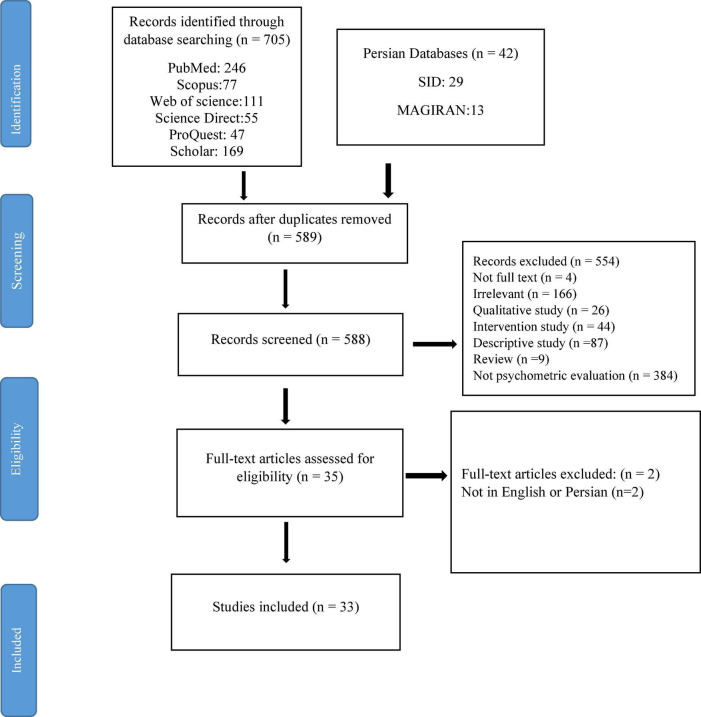
Preferred reporting items for systematic reviews and meta-analyses flowchart.

The initial search yielded 747 articles, 77 were from Scopus, 246 were from PubMed, 111 were from Web of Science, 55 were from Science Direct, 169 were from Google Scholar, 47 were from ProQuest, and 42 were from Persian databases. Of the 747 articles initially identified, 33 met all the inclusion criteria. See reasons for exclusion in Figure 1.

All of the articles found by searching databases were stored in an EndNote (version X8; Thomson Reuters, New York, NY, United States) file to display duplicate results. Two authors (LH and HN) independently evaluated all articles for inclusion and exclusion. Any discrepancy between the authors was resolved through joint discussions with the third author. See the selection process schematically in Figure 1.

### Data Extraction

The data were extracted by two researchers (LH and HN) where one was an expert in statistics extracted data and another was an expert in the concept of the study. A data extraction sheet included: first author name, publication year, country, name of scale, target population, face validity, content validity, construct validity (sample size, factor extraction method, rotation methods, selection of the number of factors, name of factors, and total variance), and reliability [consistency: Cronbach’s alpha coefficient, stability: Spearman’s correlation coefficient, and Intraclass Correlation Coefficient (ICC)] (see Supplementary Table 1).

### Risk of Bias

The Consensus-based Standards for the selection of health Measurement Instruments (COSMIN) Risk of Bias checklist was used to assess this feature for each of the 33 studies. This tool includes 3 parts with 10 boxes. The first part addresses content validity and includes boxes 1 and 2. This part assesses the relevance and comprehensibility of all items with the target construct and population. Second Part with boxes 3, 4, and 5 addresses internal structure with structural validity, internal consistency, and cross-cultural validity/measurement invariance. The third part with boxes 6, 7, 8, 9, and 10 address the remaining measurement properties including reliability, measurement error, criterion validity, hypotheses testing for construct validity, and responsiveness. The third part focuses on the quality of the (sub)scale as a whole, rather than on item level (Mokkink et al., 2018).

### Quality Assessment and Data Analysis

The full text of the articles was evaluated in terms of methodological quality based on the checklist provided by COSMIN. The COSMIN checklist assesses different psychometric properties including: A = internal consistency, B = reliability, C = measurement error, D = content validity, E = structural validity, F = hypothesis testing, G = cross-cultural validity, H = criterion validity and I = responsiveness. Finally, each article was analyzed using a four-point COSMIN score. Each item was classified into four levels including “excellent” as an appropriate methodology, “good” as an adequate level of quality and insufficient relevant information, “fair” as the questionable methodological process, and “poor” as an incorrect methodological process. A methodological quality score per box is obtained by taking the lowest rating of any item in a box (“worst score counts”) (Terwee et al., 2012). Finally, Terwee’s study criteria were used to analyze the quality criteria of the measured properties (Terwee et al., 2007). The Inter-reviewer reliability was evaluated according to the Cohen’s Kappa value. Any discrepancies were resolved through discussion and consensus.

### Data Synthesis

Since a general analysis of psychometric properties is not possible, the characteristics of the available articles were used to determine the validity of the instrument.

## Results

### Study Characteristics

A total of 747 articles were found; of these 42 articles were from the Persian database and 705 articles were from English language databases. Duplicate articles were excluded and 33 articles were reminded and were evaluated using the COSMIN checklist and Terwee study criteria (see PRISMA flow chart, Figure 1).

Studies were published between 1986 and 2021; the majority of them were published between 2016 and 2020 (each year *n* = 4). One study was a doctoral thesis (Ferrara, 2019) and the 32 other articles were original and were published in journals. The majority of them were conducted in the United States (*n* = 12) (Funk and Houston, 1987; Pollock and Duffy, 1990; Bartone, 1991; Benishek, 1996; Velasco-Whetsell and Pollock, 1999; Wang, 1999; Benishek and Lopez, 2001; Benishek et al., 2005; Maddi et al., 2006; Madrigal et al., 2016; Weigold et al., 2016) after that Iran (*n* = 4) (Mohsenabadi and Fathi-Ashtiani, 2021; Soheili et al., 2021a,b; Hosseini et al., 2022), Canada (*n* = 2) (McNeil et al., 1986; Lang et al., 2003), Brazil (*n* = 1) (Solano et al., 2016), China (*n* = 1) (Wong et al., 2014), Netherlands (*n* = 2) (Gebhardt et al., 2001; Dymecka et al., 2020), Greece (*n* = 1) (Kamtsios and Karagiannopoulou, 2013), Croatia (*n* = 1) (Kardum et al., 2012), Italia (*n* = 1) (Picardi et al., 2012), Spain (*n* = 2) (Moreno-Jiménez et al., 2014; Luceño-Moreno et al., 2020), Australia (*n* = 1) (Creed et al., 2013), Sweden (*n* = 1) (Persson et al., 2016), Taiwan (*n* = 1) (Cheng et al., 2019), Japan (*n* = 1) (Yamaguchi et al., 2020), South Korea (*n* = 1) (Ko et al., 2018), and Norway (*n* = 1) (Hystad et al., 2010). Only one study was published in the Persian language (Mohsenabadi and Fathi-Ashtiani, 2021). The majority of them were focused on student (*n* = 10) after that they conducted on general population (*n* = 6), patients (*n* = 3), parents (*n* = 3), military (*n* = 3), employee (*n* = 3), health workers (*n* = 2), athletes (*n* = 2), and family caregivers (*n* = 1).

### Findings From the Risk of Bias Evaluation

Using the COSMIN Risk of bias checklist, the quality of the research manuscripts included in this review was evaluated. From 33 articles, only 45.4% of the studies (15 articles) scored “very good” on both content validity boxes. Also, only 39.3% of the studies (13 articles) scored “very good” on both internal structure boxes. The third part of the risk of bias assessment includes 4 boxes that only 4 studies reported on 3 of 4 boxes as not very good; just one study got a “very good” score in 2 boxes (Hosseini et al., 2022). Details of the risk of bias have been reported in Table 2.

**TABLE 2:** The COSMIN risk of bias checklist.

Number	References	BOX 1	BOX 2	BOX 3	BOX 4	BOX 5	BOX 6	BOX 7	BOX 8	BOX 9	BOX10
		
		PROM development	Content validity	Structural validity	Internal consistency	Cross-cultural validity \measurement invariance	Reliability	Measurement error	Criterion validity	Hypotheses testing for construct validity	Responsiveness
1	McNeil et al., 1986	Very good	Inadequate	Very good	Inadequate	-	Inadequate	Inadequate	-	Very good	
2	Funk and Houston, 1987	Very good	Inadequate	Very good	Inadequate	-	Inadequate	Inadequate	-	Very good	
3	Pollock and Duffy, 1990	Very good	Very good	Very good	Very good	-	Inadequate	Inadequate	-	Adequate	
4	Bartone, 1991	Very good	Inadequate	Doubtful	Adequate	-	Inadequate	Inadequate	-	Inadequate	-
5	Benishek, 1996	Very good	Inadequate	Very good	Doubtful	-	Inadequate	Inadequate	-	Inadequate	-
6	Wang, 1999	Very good	Inadequate	Doubtful	Doubtful	Adequate	Inadequate	Inadequate	-	Inadequate	-
7	Velasco-Whetsell and Pollock, 1999	Very good	Adequate	Inadequate	Inadequate	Adequate	Inadequate	Inadequate	-	Inadequate	-
8	Gebhardt et al., 2001	Very good	Inadequate	Very good	Doubtful	Inadequate	Inadequate	Inadequate	-	Adequate	-
9	Benishek and Lopez, 2001	Very good	Inadequate	Very good	Very good	-	Inadequate	Inadequate	-	Very good	-
10	Lang et al., 2003	Very good	Very good	Very good	Inadequate	Inadequate	Inadequate	Inadequate	-	Very good	-
11	Benishek et al., 2005	Very good	Very good	Very good	Very good	-	Inadequate	Inadequate	-	Very good	-
12	Maddi et al., 2006	Very good	Inadequate	Very good	Doubtful	-	Inadequate	Inadequate	-	Very good	-
13	Hystad et al., 2010	Very good	Inadequate	Very good	Doubtful	-	Inadequate	Inadequate	-	Inadequate	-
14	Kardum et al., 2012	Very good	Inadequate	Very good	Doubtful	-	Inadequate	Inadequate	-	Very good	-
15	Picardi et al., 2012	Very good	Adequate	Doubtful	Doubtful	Adequate	Very good	Inadequate	Doubtful	Inadequate	-
16	Kamtsios and Karagiannopoulou, 2013	Very good	Very good	Very good	Doubtful	-	Very good	Inadequate	-	Inadequate	-
17	Creed et al., 2013	Very good	Adequate	Very good	Inadequate	-	Inadequate	Inadequate	-	Very good	-
18	Moreno-Jiménez et al., 2014	Very good	Very good	Very good	Very good	Inadequate	Inadequate	Inadequate	-	Very good	-
19	Wong et al., 2014	Very good	Very good	Very good	Doubtful	Very good	Inadequate	Inadequate	-	Very good	-
		PROM development	Content validity	Structural validity	Internal consistency	Cross-cultural validity \measurement invariance	Reliability	Measurement error	Criterion validity	Hypotheses testing for construct validity	Responsiveness
20	Persson et al., 2016	Very good	Inadequate	Very good	Doubtful	Adequate	Inadequate	Inadequate	-	Very good	-
21	Weigold et al., 2016	Very good	Inadequate	Very good	Very good	-	Inadequate	Inadequate	-	Very good	-
22	Madrigal et al., 2016	Very good	Inadequate	Very good	Doubtful	-	Inadequate	Inadequate	-	Very good	-
23	Solano et al., 2016	Very good	Very good	Very good	Doubtful	Adequate	Very good	Inadequate	-	Very good	-
24	Ko et al., 2018	Very good	Very good	Very good	Very good	Adequate	Very good	Inadequate	Doubtful	Adequate	-
25	Ferrara, 2019	Very good	Adequate	Doubtful	Doubtful	-	Inadequate	Inadequate	-	Inadequate	-
26	Cheng et al., 2019	Very good	Very good	Very good	Doubtful	-	Inadequate	Inadequate	-	Very good	-
27	Yamaguchi et al., 2020	Very good	Very good	Very good	Very good	-	Inadequate	Inadequate	-	Very good	-
28	Soheili et al., 2021a	Very good	Very good	Very good	Very good	-	Inadequate	Inadequate	-	Very good	-
29	Dymecka et al., 2020	Very good	Inadequate	Very good	Very good	Adequate	Inadequate	Inadequate	Doubtful	Very good	-
30	Luceño-Moreno et al., 2020	Very good	Inadequate	Very good	Very good	-	Inadequate	Inadequate	-	Adequate	-
31	Mohsenabadi and Fathi-Ashtiani, 2021	Very good	Very good	Very good	Very good	Very good	Inadequate	Inadequate	-	Adequate	-
32	Soheili et al., 2021b	Very good	Very good	Very good	Very good	-	Inadequate	Inadequate	-	Adequate	-
33	Hosseini et al., 2022	Very good	Very good	Very good	Very good	-	Very good	Very good	-	-	Very good

### Psychometric Properties

Concerning the study design, 15 studies were conducted to develop a scale and 18 of them assessed the psychometric properties. See details of psychometric characteristics in Supplementary Table 1. These scales were different based on item number and dimensions. The minimum item number was 12 (Kardum et al., 2012; Dymecka et al., 2020; Yamaguchi et al., 2020) and the maximum was 45 (Lang et al., 2003). Also, the minimum numbers of dimensions were one in two studies (McNeil et al., 1986; Kardum et al., 2012) and one instrument had 9 dimensions (Kamtsios and Karagiannopoulou, 2013). From these 33 studies, 31 studies tested internal consistency, 16 tested test-retest reliability, two studies tested criterion validity (Ko et al., 2018; Dymecka et al., 2020), and 30 studies tested construct validity. Most of the studies evaluated internal consistency and stability using Cronbach’s alpha, but four studies evaluated stability using ICC (Picardi et al., 2012; Kamtsios and Karagiannopoulou, 2013; Solano et al., 2016; Hosseini et al., 2022). The criterion validity was tested in two studies (Ko et al., 2018; Dymecka et al., 2020). The construct validity was tested using principal components factor or principal axis factor analysis in most of the studies (*n* = 16), exploratory factor analysis (*n* = 3), and confirmatory factor analysis (CFA) was assessed in 10 studies. Five studies did not evaluate the construct validity. The total variance that is explained with these scales ranges from 32.1% to 69% and 15 studies did not report it.

### Quality Assessment

The details of the COSMIN quality assessment of 33 articles are shown in Tables 3, 4. None of these articles had “Excellent” quality in all psychometric properties.

**TABLE 3:** COSMIN quality assessment.

Number	First author (year)	BOX A Internal consistency	BOX B Reliability	BOX C Measurement error	BOX D Content validity	BOX E Structural validity	BOX F Hypothesis testing	BOX G Cross-cultural validity	BOX H Criterion validity
		1. Adequate sample size (≥ 100) 2. Calculate the internal consistency for each dimension (sub)scale 3. Cronbach’s alpha (s) between 0.70 and 0.95	1. Available at least two measurements 2. Adequate sample size (≥ 100) 3. Calculated ICC or weighted Kappa ≥ 0.70	Calculated the Standard Error of Measurement (SEM)	Assessment of the relevancy of all items to 1. The construct 2. The study population 3. The measurement Purpose 4. Experts involved in item selection	1. Perform EFA or CFA	1. Specific hypotheses were formulated 2.75% of the results are in accordance with these hypotheses	1. Describing translation process 2. Translating item forward and backward 3. Independently 4. Adequate sample size 5. Pre-testing the scale 6. Performing CFA	1. Using the gold standard 2. Correlation with gold standard is > 0.70
1	McNeil et al., 1986	1. Yes, 2. No, 3. No	1. Yes, 2. Yes, 3. No	No	No, 2. No, 3. No, 4. No	Yes	1. Yes, 2. Yes	1. No, 2. No, 3. No, 4. No, 5. No, 6. No	1. No, 2. No
2	Funk and Houston, 1987	1. No, 2. No, 3. No	1. No, 2. No, 3. No	No	1. No, 2. No, 3. No, 4. No	Yes	1. Yes, 2. Yes	1. No, 2. No, 3. No, 4. No, 5. No, 6. No	1. No, 2. No
3	Pollock and Duffy, 1990	1. Yes, 2. Yes, 3. Yes,	1. Yes, 2. Yes, 3. No	No	Yes, 2. Yes, 3. Yes, 4. Yes	Yes	1. No, 2. No	1. No, 2. No, 3. No, 4. No, 5. No, 6. No	1. No, 2. No
4	Bartone, 1991	1. Yes, 2. No, 3. Yes	1. Yes, 2. Yes, 3. No	No	No, 2. No, 3. No, 4. No	Yes	1. No, 2. No	1. No, 2. No, 3. No, 4. No, 5. No, 6. No	1. No, 2. No
5	Benishek, 1996	1. Yes, 2. Yes, 3. No	1. No, 2. No, 3. No	No	No, 2. No, 3. No, 4 . No	Yes	1. No, 2. No	1. No, 2. No, 3. No, 4. No, 5. No, 6. Yes	1. No, 2. No
6	Wang, 1999	1. Yes, 2. Yes, 3. No	1. No, 2. No, 3. No	No	No, 2. No, 3. No, 4. No	No	1. No, 2. No	1. Yes, 2. Yes, 3. Yes, 4. Yes, 5. Yes, 6. No	1. No, 2. No
7	Velasco-Whetsell and Pollock, 1999	1. No, 2. No, 3. No	1. No, 2. No, 3. No	No	Yes, 2. Yes, 3. Yes, 4. No	No	1. No, 2. No	1. Yes, 2. Yes, 3. Yes, 4. Yes, 5. Yes, 6. No	1. No, 2. No
8	Gebhardt et al., 2001	1. Yes, 2. Yes, 3. No	1. No, 2. No, 3. No	No	No, 2. No, 3. No, 4. No	Yes	1. Yes, 2. No	1. No, 2. Yes, 3. No, 4. No, 5. No, 6. No	1. No, 2. No
9	Benishek and Lopez, 2001	1. Yes, 2. Yes, 3. Yes,	1. No, 2. No, 3. No	No	No, 2. No, 3. No, 4. No	Yes	1. Yes, 2. Yes	1. No, 2. No, 3. No, 4. No, 5. No, 6. No	1. No, 2. No
10	Lang et al., 2003	1. No, 2. Yes, 3. No	1. Yes, 2. Yes, 3. No	No	Yes, 2. Yes, 3. Yes, 4. Yes	Yes	1. Yes, 2. Yes	1. Yes, 2. Yes, 3. Yes, 4. No, 5. No, 6. No	1. No, 2. No
11	Benishek et al., 2005	1. Yes, 2. Yes, 3. Yes	1. Yes, 2. Yes, 3. No	No	Yes, 2. Yes, 3. Yes, 4. Yes	Yes	1. Yes, 2. Yes	1. No, 2. No, 3. No, 4. No, 5. No, 6. No	1. No, 2. No
12	Maddi et al., 2006	1. Yes, 2. Yes, 3. No	1. No, 2. No, 3. No	No	No, 2. No, 3. No, 4. No	Yes	1. Yes, 2. Yes	1. No, 2. No, 3. No, 4. No, 5. No, 6. No	1. No, 2. No
13	Hystad et al., 2010	1. Yes, 2. Yes, 3. No	1. No, 2. No, 3. No	No	No, 2. No, 3. No, 4. No	Yes	1. No, 2. No	1. No, 2. No, 3. No, 4. No, 5. No, 6. Yes	1. No, 2. No
14	Kardum et al., 2012	1. Yes, 2. Yes, 3. No	1. No, 2. No, 3. No	No	No, 2. No, 3. No, 4. No	Yes	1. Yes, 2. Yes	1. No, 2. No, 3. No, 4. No, 5. No, 6. Yes	1. No, 2. No
15	Picardi et al., 2012	1. Yes, 2. Yes, 3. No	1. Yes, 3. Yes, 4. Yes	No	Yes, 2. Yes, 3. Yes, 4. No	No	1. No, 2. No	1. Yes, 2. Yes, 3. Yes, 4. Yes, 5. Yes, 6. No	1. Yes, 2. No
16	Kamtsios and Karagiannopoulou, 2013	1. Yes, 2. Yes, 3. No	1. Yes, 2. Yes, 3. Yes	No	Yes, 2. Yes, 3. Yes, 4. Yes	Yes	1. No, 2. No	1. No, 2. No, 3. No, 4. No, 5. No, 6. Yes	1. No, 2. No
17	Creed et al., 2013	1. No, 2. No, 3. No	1. No, 2. No, 3. No	No	Yes, 2. Yes, 3. Yes, 4. Yes	Yes	1. Yes, 2. Yes	1. No, 2. No, 3. No, 4. No, 5. No, 6. Yes	1. No, 2. No
18	Moreno-Jiménez et al., 2014	1. Yes, 2. Yes, 3. Yes	1. Yes, 2. No, 3. No	No	Yes, 2. Yes, 3. Yes, 4. Yes	Yes	1. Yes, 2. Yes	1. Yes, 2. No, 3. No, 4. No, 5. No, 6. Yes	1. No, 2. No
19	Wong et al., 2014	1. Yes, 2. Yes, 3. No	1. No, 2. No, 3. No	No	Yes, 2. Yes, 3. Yes, 4. Yes	Yes	1. Yes, 2. Yes	1. Yes, 2. Yes, 3. Yes, 4. Yes, 5. Yes, 6. Yes	1. No, 2. No
20	Persson et al., 2016	1. Yes, 2. Yes, 3. No	1. No, 2. No, 3. No	No	No, 2. No, 3. No, 4. No	Yes	1. Yes, 2. Yes	1. Yes, 2. Yes, 3. Yes, 4. Yes, 5. No, 6. Yes	1. No, 2. No
21	Weigold et al., 2016	1. Yes, 2. Yes, 3. Yes	1. Yes, 2. Yes, 3. No	No	No, 2. No, 3. No, 4. No	Yes	1. Yes, 2. Yes	1. No, 2. No, 3. No, 4. No, 5. No, 6. No	1. No, 2. No
22	Madrigal et al., 2016	1. Yes, 2. Yes, 3. No	1. No, 2. No, 3. No	No	No, 2. No, 3. No, 4. No	Yes	1. Yes, 2. Yes	1. No, 2. No, 3. No, 4. No, 5. No, 6. No	1. No, 2. No
23	Solano et al., 2016	1. Yes, 2. Yes, 3. No	1. Yes, 2. Yes, 3. Yes	No	Yes, 2. Yes, 3. Yes, 4. Yes	Yes	1. Yes, 2. Yes	1. Yes, 2. Yes, 3. Yes, 4. Yes, 5. Yes, 6. No	1. No, 2. No
24	Ko et al., 2018	1. Yes, 2. Yes, 3. Yes	1. Yes, 2. Yes, 3. Yes	No	Yes, 2. Yes, 3. Yes, 4. Yes	Yes	1. Yes, 2. No	1. Yes, 2. Yes, 3. Yes, 4. Yes, 5. No, 6. Yes	1. Yes, 2. No
25	Ferrara, 2019	1. Yes, 2. Yes, 3. No	1. No, 2. No, 3. No	No	Yes, 2. Yes, 3. Yes, 4. No	No	1. No, 2. No	1. No, 2. No, 3. No, 4. No, 5. No, 6. No	1. No, 2. No
26	Cheng et al., 2019	1. Yes, 2. Yes, 3. No	No, 2. No, 3. No	No	Yes, 2. Yes, 3. Yes, 4. Yes	Yes	1. Yes, 2. Yes	1. No, 2. No, 3. No, 4. No, 5. No, 6. No	1. No, 2. No
27	Yamaguchi et al., 2020	1. Yes, 2. Yes, 3. Yes	No, 2. No, 3. No	No	Yes, 2. Yes, 3. Yes, 4. Yes	Yes	1. Yes, 2. Yes	1. No, 2. No, 3. No, 4. No, 5. No, 6. Yes	1. No, 2. No
28	Soheili et al., 2021a	1. Yes, 2. Yes, 3. Yes	No, 2. No, 3. No	No	Yes, 2. Yes, 3. Yes, 4. Yes	Yes	1. Yes, 2. Yes	1. No, 2. No, 3. No, 4. No, 5. No, 6. Yes	1. No, 2. No
29	Dymecka et al., 2020	1. Yes, 2. Yes, 3. Yes	No, 2. No, 3. No	No	No, 2. No, 3. No, 4. No	Yes	1. Yes, 2. Yes	1. Yes, 2. Yes, 3. Yes, 4. Yes, 5. No, 6. Yes	1. Yes, 2. No
30	Luceño-Moreno et al., 2020	1. Yes, 2. Yes, 3. Yes	No, 2. No, 3. No	No	No, 2. No, 3. No, 4. No	Yes	1. No, 2. No	1. No, 2. No, 3. No, 4. No, 5. No, 6. Yes	1. No, 2. No
31	Mohsenabadi and Fathi-Ashtiani, 2021	1. Yes, 2. Yes, 3. Yes	1. Yes, 2. Yes, 3. No	No	Yes, 2. Yes, 3. Yes, 4. Yes	Yes	1. No, 2. No	1. Yes, 2. Yes, 3. Yes, 4. Yes, 5. Yes, 6. Yes	1. No, 2. No
32	Soheili et al., 2021b	1. Yes, 2. Yes, 3. Yes	1. No, 2. No, 3. No	No	Yes, 2. Yes, 3. Yes, 4. Yes	Yes	1. Yes, 2. No	1. No, 2. No, 3. No, 4. No, 5. No, 6. No	1. No, 2. No
33	Hosseini et al., 2022	1. Yes, 2. Yes, 3. Yes	1. Yes, 2. Yes, 3. Yes	Yes	Yes, 2. Yes, 3. Yes, 4. Yes	Yes	1. No, 2. No	1. No, 2. No, 3. No, 4. No, 5. No, 6. No	1. No, 2. No

**TABLE 4:** COSMIN quality assessment.

Number	First author (year)	COSMIN boxes							
		
		BOX A Internal consistency	BOX B Reliability	BOX C Measurement error	BOX D Content validity	BOX E Structural validity	BOX F Hypothesis testing	BOX G Cross-cultural validity	BOX H Criterion validity
1	McNeil et al., 1986	Poor	Poor	Poor	Poor	Excellent	Excellent	-	-
2	Funk and Houston, 1987	Poor	Poor	Poor	Poor	Excellent	Excellent	-	-
3	Pollock and Duffy, 1990	Excellent	Poor	Poor	Excellent	Excellent	Good	-	-
4	Bartone, 1991	Good	Poor	Poor	Poor	Fair	Poor	-	-
5	Benishek, 1996	Fair	Poor	Poor	Poor	Excellent	Poor	-	-
6	Wang, 1999	Fair	Poor	Poor	Poor	Fair	Poor	Good	-
7	Velasco-Whetsell and Pollock, 1999	Poor	Poor	Poor	Good	Fair	Poor	Good	-
8	Gebhardt et al., 2001	Fair	Poor	Poor	Poor	Excellent	Good	Poor	-
9	Benishek and Lopez, 2001	Excellent	Poor	Poor	Poor	Excellent	Excellent	-	-
10	Lang et al., 2003	Poor	Poor	Poor	Excellent	Excellent	Excellent	Poor	-
11	Benishek et al., 2005	Excellent	Poor	Poor	Excellent	Excellent	Excellent	-	-
12	Maddi et al., 2006	Fair	Poor	Poor	Poor	Excellent	Excellent	-	-
13	Hystad et al., 2010	Fair	Poor	Poor	Poor	Excellent	Poor	-	-
14	Kardum et al., 2012	Fair	Poor	Poor	Poor	Excellent	Excellent	-	
15	Picardi et al., 2012	Fair	Excellent	Poor	Good	Fair	Poor	Good	Fair
16	Kamtsios and Karagiannopoulou, 2013	Fair	Excellent	Poor	Excellent	Excellent	Poor	-	-
17	Creed et al., 2013	Poor	Poor	Poor	Good	Excellent	Excellent	-	-
18	Moreno-Jiménez et al., 2014	Excellent	Poor	Poor	Excellent	Excellent	Excellent	Poor	-
19	Wong et al., 2014	Fair	Poor	Poor	Excellent	Excellent	Excellent	Excellent	-
20	Persson et al., 2016	Fair	Poor	Poor	Poor	Excellent	Excellent	Good	-
21	Weigold et al., 2016	Excellent	Poor	Poor	Poor	Excellent	Excellent	-	-
22	Madrigal et al., 2016	Fair	Poor	Poor	Poor	Excellent	Excellent	-	-
23	Solano et al., 2016	Fair	Excellent	Poor	Excellent	Excellent	Excellent	Good	-
24	Ko et al., 2018	Excellent	Excellent	Poor	Excellent	Excellent	Good	Good	Fair
25	Ferrara, 2019	Fair	Poor	Poor	Good	Fair	Poor	-	-
26	Cheng et al., 2019	Fair	Poor	Poor	Excellent	Excellent	Excellent	-	-
27	Yamaguchi et al., 2020	Excellent	Poor	Poor	Excellent	Excellent	Excellent	-	-
28	Soheili et al., 2021a	Excellent	Poor	Poor	Excellent	Excellent	Excellent	-	-
29	Dymecka et al., 2020	Excellent	Poor	Poor	Poor	Excellent	Excellent	Good	Fair
30	Luceño-Moreno et al., 2020	Excellent	Poor	Poor	Poor	Excellent	Good	-	
31	Mohsenabadi and Fathi-Ashtiani, 2021	Excellent	Poor	Poor	Excellent	Excellent	Good	Excellent	-
32	Soheili et al., 2021b	Excellent	Poor	Poor	Excellent	Excellent	Good	-	-
33	Hosseini et al., 2022	Excellent	Excellent	Excellent	Excellent	Excellent	Poor	-	-

### BOX A—Internal Consistency

The interrelatedness among the items of each scale was determined by measuring internal consistency. The main quality criteria to evaluate internal consistency are as follows: (1) adequate sample size (seven per items and > 100), (2) calculating Cronbach’s alpha (s) for each dimension separately, and (3) Cronbach’s alpha (s) between 0.70 and 0.95 (Terwee et al., 2007). Based on these criteria 13 studies were evaluated as “Excellent,” one study was “good” because it did not calculate alpha for each dimension/subscale separately (Bartone, 1991). Three studies did not evaluate internal consistency (Funk and Houston, 1987; Velasco-Whetsell and Pollock, 1999; Creed et al., 2013) and were deemed of “poor” quality. Two studies were evaluated as “poor” because did not meet two of the three criteria (McNeil et al., 1986; Lang et al., 2003). Finally, 14 studies were evaluated as “fair” because their Cronbach’s alpha (s) were < 0.70 or > 0.95.

### BOX B—Reliability

Reliability was used to show that score did not change by repeating the measurement with three methods: (1) test-retest for overtime, (2) inter-rater for measuring by different persons on the same occasion, and (3) intra-rater for measuring by the same persons (i.e., raters or responders) on different occasions. The main quality criteria to evaluate reliability are ICC or weighted Kappa ≥ 0.70 (Terwee et al., 2007). Five studies were evaluated as “Excellent,” (Picardi et al., 2012; Kamtsios and Karagiannopoulou, 2013; Solano et al., 2016; Ko et al., 2018; Hosseini et al., 2022), eight studies were evaluated as “poor” because they did not report ICC or Kappa value; and 20 studies did not evaluated reliability and were deemed of “poor” quality.

### BOX C—Measurement Error

The means of measurement error is the systematic and random error of a score that cannot be attributed to true changes in the construct reported by the Standard Error of Measurement (SEM). Just one study reported measurement errors (Hosseini et al., 2022).

### BOX D—Content Validity

Content validity is defined as “the content of the scale items reflects the structure we intend to measure.” The quality criteria to evaluate the content validity are assessment of the relevancy of all items to the construct, the study population, the measurement purpose, and experts involved in item selection. 15 studies did not report content validity and they were evaluated as “poor.” Four studies did not mention who was involved in content validity and they were evaluated as “good” (Velasco-Whetsell and Pollock, 1999; Picardi et al., 2012; Creed et al., 2013; Ferrara, 2019) and 14 of others were evaluated as “Excellent.”

### BOX E—Structural Validity

Structural validity refers to the degree to which the scores obtained from the scale reflect sufficient dimensions of the construct. Main quality criteria that show this feature are performing factor analysis by FEA or CFA. In this review, five studies did not report factor analysis and were evaluated as “fair” (Bartone, 1991; Velasco-Whetsell and Pollock, 1999; Wang, 1999; Picardi et al., 2012; Ferrara, 2019). Other studies were evaluated as “Excellent.”

### BOX F—Hypothesis Testing

Based on the COSMIN checklist, the purpose of hypothesis testing is the same as construct validity. The main quality criteria that show this feature are formulating specific hypotheses and at least 75% of the results are in accordance with these hypotheses. Nine studies did not report about construct validity and were scored as “poor” (Bartone, 1991; Benishek, 1996; Velasco-Whetsell and Pollock, 1999; Wang, 1999; Hystad et al., 2010; Picardi et al., 2012; Kamtsios and Karagiannopoulou, 2013; Ferrara, 2019; Hosseini et al., 2022), six studies did not report enough results and were evaluated as “good” (Pollock and Duffy, 1990; Gebhardt et al., 2001; Ko et al., 2018; Luceño-Moreno et al., 2020; Mohsenabadi and Fathi-Ashtiani, 2021; Soheili et al., 2021a) and the 18 remaining studies reported construct validity with complete details and were scored as “excellent.”

### BOX G—Cross-Cultural Validity

According to the COSMIN checklist, cross-cultural research refers to the ability to translate items to reflect the original version of the scale items. The main criteria for assessing these features are as follows: (1) describing the translation process, (2) translating items forward and backward, (3) independently, (4) adequate sample size, (5) pre-testing the scale, and (6) performing Confirmatory Factor Analysis (CFA). Three studies had mentioned that they translated the scale but they did not report the details and were considered “poor” (Gebhardt et al., 2001; Lang et al., 2003; Moreno-Jiménez et al., 2014). Seven studies were evaluated as “good” (Velasco-Whetsell and Pollock, 1999; Wang, 1999; Picardi et al., 2012; Persson et al., 2016; Solano et al., 2016; Ko et al., 2018; Dymecka et al., 2020) because they did not perform CFA or pre-testing. Two studies reported cross-cultural processes with details and they were evaluated as “excellent” (Wong et al., 2014; Mohsenabadi and Fathi-Ashtiani, 2021).

### BOX H—Criterion Validity

Criterion validity indicates the degree to which the scores of the scale are an adequate reflection of a “gold standard”. The main quality criteria are using the gold standard (having convincing arguments) and the current scale correlates > 0.70 with this gold standard. Three studies had reported the criterion validity as follows: (1) Angelo Picardi et al. performed criterion validity by assessing the correlation between the 15-item Dispositional Resilience Scale (DRS-15) and Psychological Well-Being Scale (as gold standard) (Picardi et al., 2012). (2) Kim et al. reported the criterion validity by assessing correlation among DRS-15, the Korean version of the Center for Epidemiological Studies-Depression Scale (KCES-D), and the Korean Resilience Questionnaire (KRQ-53) (Ko et al., 2018). (3) Dymecka et al. also reported the criterion validity by assessing correlation among health-related hardiness scale (HRHS), Sense of coherence, Self-efficacy, Acceptance of illness, and Psychological resilience (Dymecka et al., 2020). Since the scales that they had chosen were not the gold standard and the correlation between scales was not > 0.70, these studies were evaluated as “fair.” It is noteworthy that the responsiveness categories were not analyzed, because there were no results related to that.

## Discussion

This study has evaluated the psychometric properties of 33 scales about hardiness using the COSMIN checklist. The salient findings from this study include that no studies have an “Excellent” score for all of the quality criteria of psychometric properties. Therefore, there is no robust and valid single scale for measuring the hardiness concept yet.

This systemic review evaluated all the studies related to psychometric properties about hardiness conducted in different fields, different target populations, different publication times, and countries. Since present life is associated with multiple fast-paced changes and stressful circumstances, individuals in every stage of life, field, and situations need to be able to develop hardiness to face life’s difficulties. The results show that the development of scales for hardiness was conducted for any age group from children to older adults. Also, different situations were considered such as students (Benishek and Lopez, 2001; Benishek et al., 2005; Creed et al., 2013; Kamtsios and Karagiannopoulou, 2013; Cheng et al., 2019; Ferrara, 2019; Soheili et al., 2021a), athletes (Yamaguchi et al., 2020), patients (Pollock and Duffy, 1990), general population (McNeil et al., 1986; Funk and Houston, 1987; Bartone, 1991; Maddi et al., 2006; Hystad et al., 2010), parents (Lang et al., 2003; Soheili et al., 2021b), employees (Moreno-Jiménez et al., 2014), and family caregivers (Hosseini et al., 2022). Therefore, some studies were specific for a group of people with a specific situation and some of them were general. As results show, seven scales were developed for students; it may be because students are likely to experience stress and struggle and have had less opportunity to develop hardiness (Cheng et al., 2019). It should be noted that the *Dispositional Resilience Scale (DRS-15)* and *The Personal Views Survey (PVS), PVS II, PVS III, and PVS III-R* are the most frequently used scales and they were translated and assessed in several languages (Hystad et al., 2010; Wong et al., 2014; Madrigal et al., 2016; Solano et al., 2016; Ko et al., 2018; Mohsenabadi and Fathi-Ashtiani, 2021). The newest scale was the “family caregivers’ hardiness scale” for family caregivers of patients with Alzheimer’s disease (Hosseini et al., 2022).

The dimensions of all scales could be categorized into three themes as designated by Kobasa such as commitment, control, and challenge. Dimension of commitment refers to the tendency toward involvement in the situation as opposed to isolation and explains variances that ranged from 8.92 (Madrigal et al., 2016) to 38.91% (Kamtsios and Karagiannopoulou, 2013) in these studies. The Control dimension refers to belief in the effectiveness of effort on results even in stressful situations. This dimension explains the largest proportion of total explained variance of hardiness in some studies (Pollock and Duffy, 1990; Solano et al., 2016; Yamaguchi et al., 2020). The final dimension is the challenge that refers to perceiving life challenges as a normal part of life and trying to turn them into learning opportunities. This dimension also explains the largest proportion of total explained variance of hardiness in some studies (Hystad et al., 2010; Moreno-Jiménez et al., 2014; Madrigal et al., 2016). The most dimension related to Kamtsios et al. with nine factors of which six factors related to commitment, two factors related to challenging and one factor related to the control dimension (Kamtsios and Karagiannopoulou, 2013).

Since factor extraction uses for raising the explained variance with classifying items into a minimum number of factors, most studies explained total variance ≤ 50%; so that the maximum total explained variance is 68.9% related to one study with two factors (Funk and Houston, 1987), and Soheili et al. with 65.75% total variance with three factors (Soheili et al., 2021a). Also, the minimum variance explained according to the study by Pollok (32.1%) reported two factors that measured the effect of hardiness in an individual with an actual health problem (Pollock and Duffy, 1990).

Because the COSMIN checklist is the only standard tool for evaluating the quality of development and psychometric studies. It should be noted that this tool does not report the overall quality scores, because the psychometric properties items are not equal (Terwee et al., 2007). It should be noted that some studies did not report the essential information about psychometric properties clearly and they got a score “poor.” Therefore, a low-quality assessment of a scale does not indicate that this scale is inappropriate. In terms of quality, it should also be noted that the quality of more recent articles was better than older publications. This may be due to the development of guidelines by journals for writing and new statistical methods for psychometric evaluation of scales. Another noteworthy point is that most of the studies failed to report face validity, stability, measurement error, and an evaluation of responsiveness, but the newest scale designed in 2022 for family caregivers of patients with Alzheimer’s disease has all of these features.

In sum, despite the development of tool guidelines for writing and new statistical methods for psychometric evaluation of scales, each scale has at least one “Poor” psychometric property. Therefore, it is recommended that the COSMIN checklist is used for developing and accessing psychometric properties of scales to provide high-quality scales and future studies should consider features recommended by the COSMIN checklist such as face validity, stability, measurement error, and responsiveness when evaluating the psychometric properties of scales.

Finally based on the results of this systematic review, the highest methodological quality among translation and psychometric studies was the “Korean version of the 15-item Dispositional Resilience Scale” by the Ko et al. study with four boxes of COSMIN checklist scored as “Excellent,” two boxes “Good,” and one box “Fair” (Ko et al., 2018). Also, the highest methodological quality among development studies was the “family caregivers’ hardiness scale” in Hosseini et al. study (Hosseini et al., 2022) with five important boxes of the COSMIN checklist scored as “Excellent,” after that the “Occupational Hardiness Questionnaire” in Moreno-Jiménez et al. study (Moreno-Jiménez et al., 2014), “Japanese Athletic Hardiness Scale” in Yamaguchi et al. study (Yamaguchi et al., 2020), and “Children’s Hardiness Scale” in Soheili et al. study (Soheili et al., 2021b) with four boxes of COSMIN checklist scored as “Excellent.”

### Study Limitations

One of the important limitations was lack of access to the full text of the four articles (McCubbin, 1987; Godoy-Izquierdo and Godoy, 2003; Wiedebusch et al., 2007; Grau-Valdes et al., 2020) and lack of assessing two related studies. Because they were in language other than English or Persian (Madrigal et al., 2016; Serrato, 2017).

### Study Strength

Hardiness is an important psychological characteristic to deal effectively with stressful situations and reduces the negative physical and psychological effects. Since hardiness can be taught to individuals, knowing which scale has strong validity and reliability characteristics is essential to properly measure this concept. This is the first study that evaluated all scales designed since the introduction of this. Therefore, the findings of this study can help researchers choose the best scale to measure this concept accurately.

### Implication

The results of this study can help nurses, researchers, psychologists, health workers, and other decision-makers to identify the best scale concerning quality and psychometric properties.

## Conclusion

This systematic review provides information about the quality of 33 studies that assessed the psychometric properties of hardiness in various individuals in different stressful situations using the COSMIN checklist. Based on the study results, among developed scales, the “family caregivers’ hardiness scale,” “Japanese Athletic Hardiness Scale,” the “Occupational Hardiness Questionnaire,” and “Children’s Hardiness Scale” are the best for assessing hardiness in family caregivers, athletes, employees and children. In addition, the Dispositional Resilience Scale (DRS-15) and The Personal Views Survey (PVS III-R) are the most frequently used scales with suitable features for measuring hardiness in the general population.

## Data Availability Statement

The original contributions presented in the study are included in the article/supplementary material, further inquiries can be directed to the corresponding author/s.

## Author Contributions

LH and HSN designed the study protocol. LH, MA, and HSN searched the data bases and selected the suitable studies. LH and EF wrote the manuscript. All authors approved the final format of manuscript for publication.

## Conflict of Interest

The authors declare that the research was conducted in the absence of any commercial or financial relationships that could be construed as a potential conflict of interest.

## Publisher’s Note

All claims expressed in this article are solely those of the authors and do not necessarily represent those of their affiliated organizations, or those of the publisher, the editors and the reviewers. Any product that may be evaluated in this article, or claim that may be made by its manufacturer, is not guaranteed or endorsed by the publisher.

## Footnotes

1
https://www.sid.ir/

2
http://www.magiran.com/

## References

[B1] Abdollahi A. Hosseinian S. Zamanshoar E. Beh-Pajooh A. Carlbring P. (2018). The moderating effect of hardiness on the relationships between problem-solving skills and perceived stress with suicidal ideation in nursing students. *Studia Psychol.* 60 30–41. 10.21909/sp.2018.01.750

[B2] Bartone P. T. (1991). Development and validation of a short hardiness measure. *Paper Presented at the Annual Convention of the American Psychological Society*, (Washington, DC: APA).

[B3] Benishek L. A. (1996). Evaluation of the factor structure underlying two measures of hardiness. *Assessment* 3 423–435. 10.1177/107319119600300408

[B4] Benishek L. A. Feldman J. M. Shipon R. W. Mecham S. D. Lopez F. G. (2005). Development and evaluation of the revised academic hardiness scale. *J. Career Assess.* 13 59–76. 10.1177/1069072704270274

[B5] Benishek L. A. Lopez F. G. (2001). Development and initial validation of a measure of academic hardiness. *J. Career Assess.* 9 333–352. 10.1177/106907270100900402

[B6] Bigalke K. L. (2015). *Coping, Hardiness, and Parental Stress in Parents of Children Diagnosed with Cancer.* Hattiesburg: The University of Southern Mississippi.

[B7] Bonanno G. A. (2004). Have we underestimated the human capacity to thrive after extremely aversive events. *Am. Psychol.* 59 20–28. 10.1037/0003-066X.59.1.20 14736317

[B8] Chan D. W. (2000). Dimensionality of hardiness and its role in the stress-distress relationship among Chinese adolescents in Hong Kong. *J. Youth Adolesc.* 29 147–161. 10.1023/a:1005100531194

[B9] Cheng Y.-H. Tsai C.-C. Liang J.-C. (2019). Academic hardiness and academic self-efficacy in graduate studies. *High. Educ. Res. Dev.* 38 907–921. 10.1080/07294360.2019.1612858

[B10] Creed P. A. Conlon E. G. Dhaliwal K. (2013). Revisiting the academic hardiness scale: revision and revalidation. *J. Career Assess.* 21 537–554. 10.1177/1069072712475285

[B11] Dymecka J. Bidzan-Bluma I. Bidzan M. Borucka-Kotwica A. Atroszko P. Bidzan M. (2020). Validity and reliability of the Polish adaptation of the Health-Related Hardiness Scale – the first confirmatory factor analysis results for a commonly used scale. *Health Psychol. Rep.* 8, 248–262. 10.5114/hpr.2020.95746

[B12] Efimova O. I. Grinenko A. V. Kalinina N. V. Miroshkin D. V. Bazhdanova Y. V. Oshchepkov A. A. (2019). Personality hardiness as a factor determining the interaction of a person with the environment (psychological and ecological aspects). *Ekoloji Dergisi* 28 563–569.

[B13] Ferrara S. (2019). *An Initial Development of a Hardiness Scale for Elementary School Students.* Harrisonburg: James Madison University.

[B14] Funk S. C. Houston B. K. (1987). A critical analysis of the Hardiness Scale’s validity and utility. *J. Pers. Soc. Psychol.* 53:572. 10.1037/0022-3514.53.3.572

[B15] Gebhardt W. Van der Doef M. Paul L. (2001). The Revised Health Hardiness Inventory (RHHI-24): psychometric properties and relationship with self-reported health and health behavior in two Dutch samples. *Health Educ. Res.* 16 579–592. 10.1093/her/16.5.579 11675805

[B16] Godoy-Izquierdo D. Godoy J. (2003). Psychometric properties of the Spanish version of the hardiness scale (personal views survey; PVS). *Psicol. Conductual* 12 43–78.

[B17] Grau-Valdes Y. Oliva-Hernandez I. Rojas-Ricardo L. Grau-Abalo J. A. Martinez-Rodriguez L. (2020). Psychometric properties of the Hardiness Questionnaire (non-work version) in the Cuban population. *Ter. Psicol.* 38 153–167.

[B18] Green S. Grant A. M. Rynsaardt J. (2020). “Evidence-based life coaching for senior high school students: building hardiness and hope,” in *Coaching Researched: A Coaching Psychology Reader*, eds Passmore J. Tee D. (Hoboken, NJ: John Wiley & Sons), 257–268. 10.1002/9781119656913.ch13

[B19] Hosseini L. Sharif Nia H. Farahani M. A. (2021). Hardiness in family caregivers during caring from persons with Alzheimer’s disease: a deductive content analysis Study. *Front. Psychiatry* 12:770717. 10.3389/fpsyt.2021.770717 35069280 PMC8766820

[B20] Hosseini L. SharifNia H. Farahani M. A. (2022). Development and psychometric evaluation of family caregivers’ hardiness scale: a sequential exploratory mixed-method study. *Front. Psychol.* 13:807049. 10.3389/fpsyg.2022.807049 35432109 PMC9010881

[B21] Hystad S. W. Eid J. Johnsen B. H. Laberg J. C. Thomas Bartone P. (2010). Psychometric properties of the revised Norwegian dispositional resilience (hardiness) scale. *Scand. J. Psychol.* 51 237–245. 10.1111/j.1467-9450.2009.00759.x 20028488

[B22] Kamtsios S. Karagiannopoulou E. (2013). The development of a questionnaire on academic hardiness for late elementary school children. *Int. J. Educ. Res.* 58 69–78. 10.5964/ejop.v12i1.997 27247692 PMC4873066

[B23] Kardum I. Hudek-Knežević J. Krapić N. (2012). The structure of hardiness, its measurement invariance across gender and relationships with personality traits and mental health outcomes. *Psihologijske Teme* 21 487–507.

[B24] Ko E. Kim H. Y. Bartone P. T. Kang H. S. (2018). Reliability and validity of the Korean version of the 15-item Dispositional Resilience Scale. *Psychol. Health Med.* 23 (Suppl. 1), 1287–1298. 10.1080/13548506.2017.1417612 29280390

[B25] Kobasa S. C. (1979). Stressful life events, personality, and health: an inquiry into hardiness. *J. Pers. Soc. Psychol.* 37:1. 10.1037//0022-3514.37.1.1 458548

[B26] Lang A. Goulet C. Amsel R. (2003). Lang and Goulet hardiness scale: development and testing on bereaved parents following the death of their fetus/infant. *Death Stud.* 27 851–880. 10.1080/716100345 14610777

[B27] Luceño-Moreno L. Talavera-Velasco B. Jaén-Díaz M. Martín-García J. (2020). Hardy personality assessment: validating the occupational hardiness questionnaire in police officers. *Prof. Psychol. Res. Pract.* 51:297. 10.1037/pro0000285

[B28] Maddi S. R. (2004). Hardiness: an operationalization of existential courage. *J. Hum. Psychol.* 44 279–298. 10.1177/0022167804266101

[B29] Maddi S. R. (2007). “The story of hardiness: twenty years of theorizing, research, and practice,” in *The Praeger Handbook on Stress and Coping*, eds Monat A. Lazarus R. S. Reevy G. M. (Owings Mills, MD: Praeger).

[B30] Maddi S. R. Harvey R. H. Khoshaba D. M. Lu J. L. Persico M. Brow M. (2006). The personality construct of hardiness, III: relationships with repression, innovativeness, authoritarianism, and performance. *J. Pers. Soc. Psychol.* 74 575–598. 10.1111/j.1467-6494.2006.00385.x 16529587

[B31] Maddi S. R. Khoshaba D. M. (2005). *Resilience at Work: How to Succeed No Matter What Life Throws at You.* New York, NY: Amacom Books.

[B32] Madrigal L. Gill D. L. Eskridge K. M. (2016). *Examining the Reliability, Validity and Factor Structure of the DRS-15 with College Athletes.* Abingdon: Athletic Performance Research.

[B33] McCubbin M. A. (1987). *Family Hardiness Index.* Madison, WI: University of Wisconsin.

[B34] McNeil K. Kozma A. Stones M. Hannah E. (1986). Measurement of psychological hardiness in older adults. *Can. J. Aging* 5 43–48. 10.1017/s0714980800005006

[B35] Moher D. Liberati A. Tetzlaff J. Altman D. G. Group T. P. (2009). Preferred reporting items for systematic reviews and meta-analyses: the PRISMA statement. *PLoS Med.* 6:e1000097. 10.1371/journal.pmed.1000097 19621072 PMC2707599

[B36] Mohsenabadi H. Fathi-Ashtiani A. (2021). Psychometric properties of the persian version of the dispositional resiliency scale: a brief hardiness measurement scale. *J. Mil. Med.* 23 338–348.

[B37] Mokkink L. B. De Vet H. C. Prinsen C. A. Patrick D. L. Alonso J. Bouter L. M. (2018). COSMIN risk of bias checklist for systematic reviews of patient-reported outcome measures. *Qual. Life Res.* 27 1171–1179. 10.1007/s11136-017-1765-4 29260445 PMC5891552

[B38] Moreno-Jiménez B. Rodríguez-Muñoz A. Hernández E. G. Blanco L. M. (2014). Desarrollo y validación del Cuestionario Ocupacional de Resistencia. *Psicothema* 26, 207–214. 10.7334/psicothema2013.49 24755022

[B39] Mund P. (2017). Hardiness and culture: a study with reference to 3 Cs of Kobasa. *Int. Res. J. Manag. IT Soc. Sci.* 4 152–159.

[B40] Persson C. Benzein E. Årestedt K. (2016). Assessing family resources: validation of the S wedish version of the F amily H ardiness I ndex. *Scand. J. Caring Sci.* 30 845–855. 10.1111/scs.12313 26766613

[B41] Picardi A. Bartone P. T. Querci R. Bitetti D. Tarsitani L. Roselli V. (2012). Development and validation of the Italian version of the 15-item dispositional resilience scale. *Riv. Psichiatr.* 47 231–237. 10.1708/1128.12446 22825439

[B42] Pollock S. E. Duffy M. E. (1990). The health-related hardiness scale: development and psychometric analysis. *Nurs. Res.* 39 218–222. 2367202

[B43] Serrato L. (2017). Propiedades psicométricas del cuestionario construido para evaluar personalidad resistente en deportistas (PER-D). *Cuadernos Psicol. Deporte* 17 25–34.

[B44] Soheili F. Hosseinian S. Abdollahi A. (2021a). Development and initial validation of the children’s hardiness scale. *Psychol. Rep.* 124 1932–1949. 10.1177/0033294120945175 32731798

[B45] Soheili F. Hosseinian S. Abdollahi A. (2021b). Development and initial validation of the hardiness based parenting behaviors questionnaire (HBPBQ). *Curr. Psychol.* 10.1007/s12144-021-01673-z 32731798

[B46] Solano J. P. C. Bracher E. S. B. Faisal-Cury A. Ashmawi H. A. Carmona M. J. C. Lotufo F. (2016). Factor structure and psychometric properties of the Dispositional Resilience Scale among Brazilian adult patients. *Arquivos Neuro Psiquiatr.* 74 1014–1020. 10.1590/0004-282X20160148 27992001

[B47] Sharif Nia H. S. She L. Rasiah R. Fomani F. K. Kaveh O. Sharif S. P. (2021). Psychometrics of persian version of the ageism survey among an iranian older adult population during COVID-19 pandemic. *Front. Public Health* 9:683291. 10.3389/fpubh.2021.683291 34869136 PMC8637902

[B48] Terwee C. B. Bot S. D. de Boer M. R. van der Windt D. A. Knol D. L. Dekker J. (2007). Quality criteria were proposed for measurement properties of health status questionnaires. *J. Clin. Epidemiol.* 60 34–42. 10.1016/j.jclinepi.2006.03.012 17161752

[B49] Terwee C. B. Mokkink L. B. Knol D. L. Ostelo R. W. Bouter L. M. de Vet H. C. (2012). Rating the methodological quality in systematic reviews of studies on measurement properties: a scoring system for the COSMIN checklist. *Qual. Life Res.* 21 651–657. 10.1007/s11136-011-9960-1 21732199 PMC3323819

[B50] Velasco-Whetsell M. Pollock S. E. (1999). The health-related hardiness scale: spanish language equivalence and translation. *Holistic Nurs. Pract.* 13 35–43. 10.1097/00004650-199904000-00007 10418384

[B51] Wagnild G. Young H. (1988). *Hardiness among Elderly Women.* San Jose, CA: ERIC.

[B52] Wang J. F. (1999). Verification of the health-related hardiness scale: cross-cultural analysis. *Holistic Nurs. Pract.* 13 44–52. 10.1097/00004650-199904000-00008 10418385

[B53] Weigold I. K. Weigold A. Kim S. Drakeford N. M. Dykema S. A. (2016). Assessment of the psychometric properties of the revised academic hardiness scale in college student samples. *Psychol. Assess.* 28:1207. 10.1037/pas0000255 26653051

[B54] Wiedebusch S. McCubbin M. Muthny F. (2007). The Family Hardiness Index in German adaptation (FHI-D)-a questionnaire for the assessment of family resiliency. *Pravent. Rehabil.* 19:74. 10.5414/prp19074

[B55] Wong J. Y.-H. Fong D. Y.-T. Choi A. W.-M. Chan C. K.-Y. Tiwari A. Chan K. L. (2014). Transcultural and psychometric validation of the Dispositional Resilience Scale (DRS-15) in Chinese adult women. *Qual. Life Res.* 23 2489–2494. 10.1007/s11136-014-0713-9 24894382

[B56] Yamaguchi S. Kawata Y. Nakamura M. Murofushi Y. Hirosawa M. Shibata N. (2020). Development of the revised japanese athletic hardiness scale for University Athletes. *Jpn. J. Appl. Psychol.* 46 158–166.

