# Supplementary material for: Evaluation of Psychometric Properties of Hardiness Scales: A Systematic Review
Source: Front Psychol. 2022 Jun 1;13:840187. doi: 10.3389/fpsyg.2022.840187 (PMC9199987; doi:10.3389/fpsyg.2022.840187)
Supplement: Supplementary file 1 [file Table_1.docx]

**Supplementary Table 1: PSYCHOMETRIC CHARACTERISTICS OF INCLUDED STUDIES (n=33)**

| **Number** | **First author (year)** | **Scale** | **Country** | **Target population** | **Face validity** | **Content validity** | **Construct validity** | | | | | **Reliability** | |
| --- | --- | --- | --- | --- | --- | --- | --- | --- | --- | --- | --- | --- | --- |
|  |  |  |  |  |  |  | **Sample size** | **Factor extraction method (rotation)** | **Selection of the number of factors** | **Name of factor** | **Total variance (%)** | **Consistency** | **Stability** |
|  | Kevin McNeil (1986) | Psychometrics of short 20-item form of the hardiness  measure | Canada | Older adults aged over 50  years | pilot research | - | 223 | PCA (varimax) | Eigenvalues ≥1 | One Dimension  20 items | 49% | α=0.67 | α=0.63 |
|  | B. Kent Houston (1987) | Hardiness Subscales (Alienation from self, Alienation from work, Security, Powerlessness, External locus of control) | U.S.A | Students | - | - | 120 | PCA (Orthogonal) | Eigenvalues ≥1 | D1  D2 | 68.9% | - | - |
|  | Pollock (1990) | Health-related hardiness scale (HRHS) | U.S.A | Patients with diabetes (205), MS (124), HTN (36), RA(24) | 53 graduated nursing students  3 items added | Three expert  (ICC) | 389 | PCA (oblimin) | Scree plot  Eigenvalues  KMO: 0.87  Bartlett test: (p<.001) | D1: challenge/commitment (20 items)  D2: control (1items)  34 items  6-point Likert scale | 32.1% | Total α=0.91  F1: α= 0.87  F2: α= 0.87 | Total Test-retest =0.76  F1=0.74  F2=0.78 |
|  | Paul T. Bartone (1991) | Dispositional resilience scale (DRS) | USA | Army  Military personnel | - | - | 262 | - | - | F1: Challenge  F2: Control  F3: Commitment  30 items | - | Total α=0.70 to 0.85 | - |
|  | Lois A. Benishek (1996) | evaluate the factor structure  of the PVS and the RHS | U.S.A | employees | - | - | 300 | PAFs (oblimin)  CFAs: maximum likelihood | eigenvalues ≥1  scree plot  the magnitude of the item loadings  with other resulting factors  the conceptual meaningfulness of the factors | PVS  F1: Commitment  F2: control F3: challenge  15 items  RHS  F1: Commitment  F2: control F3: challenge  36 items | PVS (49.8%)  RHS(53.4%) | PVS  Total: α=0.84  F1: α=0.77  F2: α=0.62  F3: α=0.62  RHS  Total: α=0.73  F1: α=0.72  F2: α=0.74  F3: α=0.39 | - |
|  | Janet F. Wang (1999) | Chinese version of health-related hardiness scale | U.S.A & Taiwan | Nurse | - | - | 163 American  615 Taiwanese | - |  | F1: control (14 items)  F2: commitment (7 items)  F3: challenge (13 items)  34 items  Likert type 6 -point scale | - | American nurse  F1: α=0.78  F2: α=0.65  F3: α=0.58  Taiwanese nurse F1: α=0.78  F2: α=0.63  F3: α=0.81 |  |
|  | Martha velasco-whetsell (1999) | Spanish version of health-related hardiness scale (SHRHS) | U.S.A | General population | - | A panel of experts | - | - | - | - | - | - | - |
|  | W.A. Gebhardt (2001) | Dutch Revised Health Hardiness Inventory (RHHI-24) | Netherland | General populations (N=205)  Students  (N=286) | - | - | 491 | PCA (varimax) | Factor loading ≥0.30  Internal reliability  Content of the items  Scree plot | D1: Health Value (6 items)  D2: Internal Health Locus of Control (5 items)  D3: External Health Locus of Control (7 items)  D4: Perceived Health Competence (6 items)  24 items  Likert type 5 -point scale | 38.5% | general population  D1: α=0.79  D2: α=0.66  D3: α=0.67  D4: α=0.69  Student  D1: α=0.78  D2: α=0.65  D3: α=0.58  D4: α=0.76 | - |
|  | Lois A. Benishek (2001) | Academic Hardiness Scale (AHS) | U.S.A | Students age from 14 to 19 years | - | - | 481 | PAF (oblique)  CFA (covariance  matrices, maximum likelihood, and deletion methods) | Eigenvalues  scree plot  magnitude of the item loadings  co-loadings with the other resulting factors  conceptual meaningfulness of the factors | two-factor model  F1: commitment (10 items)  F2: control (3 items) & challenge (5 items)  18 items  F1: commitment (10 items)  F2: challenge (7items)  F3: control (3 items) & commitment (3 items)  23 items | 37%  42% | Total α=0.84  F1: α= 0.85  F2: α= 0.78  F3: α= 0.69 |  |
|  | Ariella Lang  (2003) | Lang and Goulet Hardiness Scale (LGHS) | Canada | parents following the  death of their fetus/infant 2 months previously |  | 15 experts  CVI: .90 | 293 | PCA (Varimax) | Scree plot | D1: Sense of personal control (16 items)  D2: Active orientation  (13 items)  D3: Sense of meaning  (16 items)  45 items  5-point Likert scale | - | Total α=0.78  D1: α= 0.60  D2: α= 0.58  D3: α= 0.64 |  |
|  | Lois A. Benishek (2005) | Revised Academic Hardiness Scale  (RAHS) | U.S.A | Students 16 to 19 years | - | fourth  member of the research team (an original developer of the first version of the  measure) | 350 | PCA (oblique) | scree plot | D1: commitment/control-effort  D2: challenge  D3: control-affect  18-item scale  4-point response scale | 33% | Total: α=0.90  D1: α=0.91  D2: α=0.88  D3: α=0.90 | Total: α=0.86  F1: α=0.86  F2: α=0.88  F3: α=0.81 |
|  | Maddi (2006) | Personal Views Survey (PVS III-R) | U.S.A | college students and  working adults |  |  | 1239 | CFA |  | F1: commitment  F2: control  F3: challenge  18 items  4-point rating scale | - | Total α=0.80  F1: α= 0.69  F2: α= 0.57  F3: α= 0.73 |  |
|  | sigurd william hystad (2010) | dispositional  resilience (hardiness) scale | Norway | employees | - | - | 7280 | PCA (promax)  CFA ( maximum likelihood) | scree-plot test  eigenvalues  Velicer’s  minimum average partial (MAP) test  Horn’s parallel  analysis | D1: control (5 items)  D2: commitment (5 items)  D3: challenge (5 items)  15 items  4-point Likert scale | 52.61% | Total α=0.79  D1: α= 0.74  D2: α= 0.76  D3: α= 0.62 | - |
|  | Igor Kardum (2012) | 15-item Dispositional Resilience  Scale (DRS-15) | Croatia | administration, management and technical jobs | - | - | 597 | CFA (maximum likelihood) | - | One factor  12 items | - | α=0.76 | - |
|  | Angelo Picardi (2012) | Italian version of the 15-item  Dispositional Resilience Scale | Italia | non-clinical sample of adults | - | - | 150 | - | - | F1: commitment  F2: control  F3: challenge  15 items  4-point Likert scale | - | total α=0.73  F1: α=0.55  F2: α=0.46  F3: α=0.74 | ICC total=  0.75  F1:  ICC =0.73  F2:  ICC = 0.69  F3:  ICC=0.75 |
|  | Spiridon Kamtsios (2013) | Academic Hardiness Questionnaire for late elementary school children | Greece | Late elementary school children (10–12 years) | 20 target population students (10–12 years) | A panel of experts on  academic hardiness theory | 1474 | PCA (varimax)  CFA (maximum likelihood method) | factor structure coefficients of 0.40 or greater  scree-plot  eigenvalues  greater than 1.0  the presence of correlation with other resulting factor  the conceptual meaningfulness of the  factors  K.M.O. =.90  Bartlett = p < .001 | D1: commitment (4 items)  D2: control-awareness (6 items)  D3: commitment: adults’ acceptance (4 items  D4: commitment: knowledge utility (5 items)  F5: control-awareness: attempt to avoid unpleasant feelings (5 items)  F6: commitment: regulating priority to learning versus enjoyment (3 items)  D7: challenge: dealing positively with hard subjects (3 items)  D8: commitment: looking for help contributing to learning (3 items)  D9: challenge: dealing with failure in a constructive way (3 items)  36 items  4-point Likert-type scale | 55.15% | Total α=0.91  D1: α= 0.83  D2: α= 0.75  D3: α= 0.77  D4 α=0.73  D5: α= 0.69  D6: α= 0.66  D7: α= 0.66  D8: α= 0.76  D9: α= 0.63 | ICC for  the nine factors ranged from .61 to .81 |
|  | Peter A. Creed.(2013) | revised  Academic Hardiness Scale | Australia | high school students 14-17 years | - | 16 experts | 300 | CFA (maximum likelihood) | - | F1: Commitment (9 items)  F2: control (4 items)  F3: challenge (4 items)  17 items | - | - | - |
|  | Bernardo Moreno-Jiménez (2014) | Occupational Hardiness Questionnaire (OHQ) | Spain | health workers | - | four experts  CVI: .85 | 1,647 | PCA (varimax)  CFA (Maximum likelihood) | Scree plot  Eigenvalues  KMO: 0.87  Bartlett test: (p<.001) | D1: challenge  D2: commitment  D3: control  15 items  4-point Likert scale | 53.1% | Total α=0.86  D1: α= 0.80  F2: α= 0.74  F3: α= 0.76  with CFA  Total α=0.85  D1: α= 0.78  D2: α= 0.73  D3: α= 0.72 | F1:  r=0.44  F2:  r=0.43  F3:  r=0.54 |
|  | Janet Yuen-Ha Wong (2014) | Chinese version of the  15-item Dispositional Resilience Scale (C-DRS-  15) | China | women aged 18 or older | Five Chinese adult women | - | 550 | PCA (oblique)  CFA | scree plot | D1: commitment (six items)  D2: control-adaptation (six items)  D3: positivity (three items)  15 items  4-point Likert scale | - | D1: α=0.78  D2: α=0.75  D3: α=0.61 | - |
|  | Carina Persson (2016) | Family Hardiness Index | Sweden | family  members to persons with cognitive dysfunctions  (n = 95) and nursing students (n = 79) | - | - | 174 | CFA | - | Three subscale version  F1: Commitment  F2: challenge  F3: control  Four subscale version  F1: Commitment  F2: challenge  F3: control  F4: Confidence  20 items  4-point Likert scale | - | Total α=0.86  Three subscale  F1: α=0.86  F2: α=0.74  F3: α=0.48  Four subscale  F1: α=0.87  F2: α=0.70  F3: α=0.16  F4: α=0.71 | - |
|  | Ingrid K. Weigold (2016) | Psychometric Properties of the Revised Academic  Hardiness Scale | U.S.A | college students Ages ranged from 18 –58 | - | - | 889 | CFA (maximum likelihood) | - | F1: Commitment (12 items)  F2: challenge (11 items)  F3: control of Affect (10 items)  F4: Control of Effort (7 items)  40 items  4-point Likert scale | - | F1: α=0.88  F2: α=0.84  F3: α=0.84  F4: α=0.79 | 1 week  F1: α=0.78  F2: α=0.71  F3: α=0.83  F4: α=0.59  2 week  F1: α=0.83  F2: α=0.78  F3: α=0.77  F4: α=0.84  4 week  F1: α=0.86  F2: α=0.76  F3: α=0.87  F4: α=0.85  6 week  F1: α=0.36  F2: α=0.71  F3: α=0.72  F4: α=0.70 |
|  | Leilani Madrigal (2016) | DRS-15 | U.S.A | collegiate athletes from a wide range of sports | - | - | 525 | CFA  EFA (maximum  likelihood, varimax) | eigenvalues ≥1 | F1: control F2: challenge F3: Commitment  F4: Purpose  15 items  4-point Likert scale | 41.26% | Total: α=0.69  F1: α=0.67  F2: α=0.67  F3: α=0.58 | - |
|  | João Paulo Consentino Solano (2016) | Brazilian version of Dispositional Resilience Scale | Brazil | adult patients  (18 years or older) of psychiatric ambulatories | 60 patients | cultural  adaptation committee (CAC) | 575 participants | PCA (oblique) | Eigenvalues above 1. | D1: control (5items)  D2: commitment (5items)  D3: challenge (4items)  14 items  4-point Likert scale | 48% | Total:  α=0.71  D1: α=0.66  D2: α=0.72  D3: α=0.64 | Total:  ICC=0.81  F1: ICC=0.70  F2:  ICC =0.78  F3:  ICC = 0.69 |
|  | Hye Young Kim (2018) | Korean version of the  15-item Dispositional Resilience Scale (K DRS) | Korea | adults aged 20–79 years | a pilot test involving  20 Korean adults | 3 nursing professors and 1 psychology professor | 409 | CFA | - | F1: commitment  F2: control  F3: challenge  15 items  4-point Likert scale | - | Total:  α=0.85  F1: α=0.81  F2: α=0.75  F3: α=0.83 | ICC= .80 to .81 |
|  | Stephen Ferrara (2019) | hardiness scale for  children  (HSC) | U.S.A | elementary school students (2nd-5th grade) | pilot testing with volunteer  elementary school students | - | 121 | - | - | F1: Challenge (5 items)  F2: Control (6 items)  F3: Commitment (4 items)  15 items  3-point Likert scale | - | Total α=0.73  F1: α= 0.57  F2: α= 0.57  F3: α= 0.47 | - |
|  | Chin-Chung Tsai (2019) | graduate students’  academic hardiness (GSAH) | Taiwan | graduate students | - | two professors  experienced in teaching and advising graduate students | 202 | PCA (varimax) | eigenvalues ≥1  scree plot  factor loadings ≥0.5 | D1: commitment to coursework (4 items)  D2: commitment to priority setting (4 items)  D3: control of effort (3 items)  F4: control of affect on confronting difficulties (3 items)  D5: control of affect on adjusting to the situation (3 items)  D6: challenge of coursework (7  items)  D7: challenge of research innovation (3 items)  27 items  5-point Likert scale | 61.87% | Total: α=0.84  D1: α=0.73  D2: α=0.66  D3: α=0.64  D4: α=0.72  D5: α=0/75  D6: α=0/87  D7: α=0/76 | - |
|  | Shinji yamaguchi (2020) | Revised Japanese Athletic hardiness scale (AHS) | Japan | athletes | - | 5 specialists | 511 | EFA  CFA (maximum likelihood) | eigenvalues ≥1  factor loadings ≥0.5 | F1: control (4 items)  F2: challenge (4 items)  F3: commitment (4 items)  12 items  4-point Likert scale | 49.9% | F1: α= 0.80  F2: α= 0.77  F3: α= 0.75 |  |
|  | Simin Hosseinian (2020) | Children’s  Hardiness Scale (CHS) | Iran | Children within the 10-12 age | 12 children | qualitative  results (four experts)  eight experts  a 3-point Likert scale  CVR:0.75  S-CVI: 0.96  I-CVI : 0.83 | 641 | PCA (Direct Oblimin)  CFA | scree plot Eigenvalues  KMO: 0.85  Bartlett test: (p<.001) | D1: commitment (9 items)  D2: control (6 items)  D3: challenge (5 items)  20 items  4-point Likert scale | 65.75% | Total α=0.85  D1: α= 0.83  D2: α= 0.79  D3: α= 0.77 |  |
|  | Joanna Dymecka (2020) | Polish adaptation  of the Health-Related Hardiness Scale | Netherland | patients | - | - | 450 | CFA (maximum  likelihood) | factor loadings | F1: challenge/commitment (6 items)  F2: Control (6 items)  12 items  6-point Likert scale | - | Total:  α=0.75  F1: α=0.75  F2: α=0.83 | - |
|  | Lourdes Luceño-Moreno (2020) | Occupational Hardiness  Questionnaire | Spain | police officers | - | - | 212 | CFA | - | F1: challenge  F2: commitment  F3: control  15 items  4-point Likert scale | - | F1:  α=0.87  F2: α=0.81  F2: α=0.81 | - |
|  | Ali Fathi-Ashtiani (2021) | Persian Version of 15-item Dispositional Resilience  Scale (DRS-15) | Iran | Military personnel | 20 Military personnel | A group of expert | 310 | CFA | - | F1: Commitment F2: control F3: challenge  15 items  4-point Likert scale | - | Total:  α=0.93  F1: α=0.88  F2: α=0.87  F3: α=0.88 | Total:  α=0.82  F1: α=0.78  F2: α=0.88  F3: α=0.70 |
|  | Simin Hosseinian  (2021) | hardiness based parenting  behaviors questionnaire (HBPBQ) | Iran | parents of children aged between 7 and 12 | 10 parents (6 female, 4 male) | 9 panels of experts | 663  EFA (325)  CFA(338) | PCA  (Direct Oblimin)  CFA | scree plot  eigenvalue≥ 1.0  Factor loading ≥  .40 | D1: responsive interaction (15 items)  D2: empowering participation (12 items)  D3: learning-oriented support (10 items)  37items  5-point Likert scale | 55.63% | Total  α=0.91  D1: α=0.91  D2: α=0.93  D3: α=0.90 | - |
|  | Lida Hosseini  (2022) | Family caregivers’ Hardiness scale: | Iran | Family caregivers of patients with Alzheimer | 11 Family caregivers | 23 expert panel | 210 (EFA)  225  (CFA) | Maximum likelihood (Promax rotation) | Parallel analysis  Factor loading ≥  .30 | F1: religious coping (5 items)  F2: Self- management (6 items)  F3: Empathic communication  (3 items)  F4: Family affective commitment (3 items)  F5: Purposeful interaction (4 items)  21 items  5-point Likert scale | 58.72% | Total  α=0.91  F1: α=0.88  F2: α=0.88  F3: α=0.76  F4: α=0.74  F5: α=0.69 | Total ICC=0.903 |
